# Right ventricular to pulmonary artery coupling in patients with different types of aortic stenosis undergoing TAVI

**DOI:** 10.1007/s00392-024-02457-8

**Published:** 2024-05-15

**Authors:** Julius Steffen, Melanie Lux, Thomas J. Stocker, Nikolaus Kneidinger, Kornelia Löw, Philipp M. Doldi, Magda Haum, Julius Fischer, Lukas Stolz, Hans Theiss, Konstantinos Rizas, Daniel Braun, Martin Orban, Sven Peterß, Jörg Hausleiter, Steffen Massberg, Simon Deseive

**Affiliations:** 1 Department of Medicine I, LMU University Hospital, LMU Munich, Marchioninistr. 15, 81377 Munich, Germany; 2 German Centre for Cardiovascular Research (DZHK), Partner Site Munich, Munich, Germany; 3 Department of Medicine V, Comprehensive Pneumology Center (CPC-M), German Center for Lung Research (DZL), LMU University Hospital, LMU Munich, Marchioninistr. 15, 81377 Munich, Germany; 4 Department of Cardiac Surgery, LMU University Hospital, LMU Munich, Marchioninistr. 15, 81377 Munich, Germany; 5 Division of Pulmonology, Department of Internal Medicine, Medical University of Graz, Graz, Austria

**Keywords:** Right ventricular pulmonary artery coupling, Aortic stenosis, TAVI, Low-flow low-gradient, Normal-flow low-gradient, VARC-3

## Abstract

**Background:**

Right ventricular (RV) dysfunction in patients undergoing transcatheter aortic valve implantation (TAVI) for aortic stenosis (AS) has long been disregarded. We aimed to assess the predictive value of RV to pulmonary artery coupling (RV/PAc), defined as tricuspid annular plane systolic excursion to systolic pulmonary artery pressure, on mortality in different flow types of AS after TAVI.

**Methods:**

All patients undergoing TAVI for AS at our centre between 2018 and 2020 were assessed; 862 patients were analysed. The cohort was dichotomized using a ROC analysis (cut-off 0.512 mm/mmHg), into 429 patients with preserved and 433 patients with reduced RV/PAc.

**Results:**

Reduced RV/PAc was associated with male sex and a higher rate of comorbidities. Short-term VARC-3 endpoints and NYHA classes at follow-up were comparable. Reduced RV/PAc was associated with higher 2-year all-cause mortality (35.0% [30.3–39.3%] vs. 15.4% [11.9–18.7%], hazard ratio 2.5 [1.9–3.4], *p* < 0.001). Cardiovascular mortality was almost tripled. Results were consistent after statistical adjustment and in a multivariate model.

Sub-analyses of AS flow types revealed lower RV/PAc in classical and paradoxical low-flow low-gradient AS, with the majority having reduced RV/PAc (74% and 59%). RV/PAc retained its predictive value in these subgroups.

**Conclusions:**

RV dysfunction defined by low RV/PAc is a strong mortality predictor after TAVI independent of flow group. It should be incorporated in future TAVI risk assessment.

**Graphical abstract:**

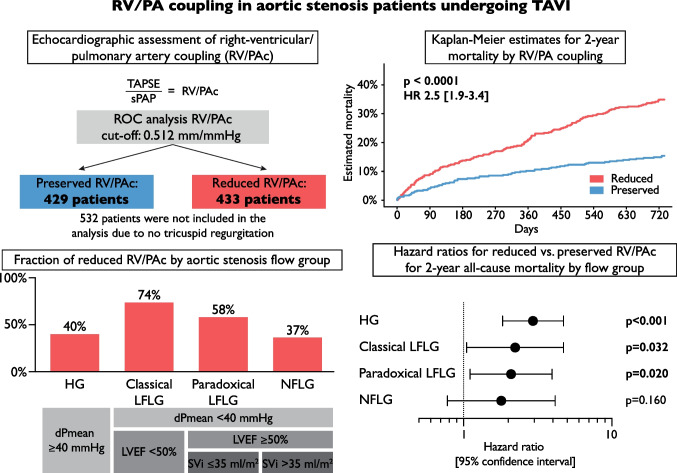

**Supplementary Information:**

The online version contains supplementary material available at 10.1007/s00392-024-02457-8.

## Introduction

Transcatheter aortic valve implantation (TAVI) has become the standard therapy for many patients with symptomatic aortic stenosis (AS) and high surgical risk [1, 2]. Extensive research is being conducted to better understand the prognosis of AS patients after TAVI and, thus, improve patient selection. This includes the identification of different sub-entities with low gradients, which are often linked to comorbidities such as ischemic cardiomyopathy, mitral regurgitation, diastolic dysfunction, or atrial fibrillation [3, 4]. In this context, right ventricular (RV) dysfunction has long been disregarded. Cardiac damage from AS might progress to the right heart, resulting in RV remodelling or dilation, potentially disimproving prognosis [5, 6]. However, this concept of AS-induced cardiac damage has also been critically discussed [4] and RV dysfunction might be an independent entity in some patients.

Right ventricular to pulmonary artery coupling (RV/PAc) is a parameter of RV function. It describes the link of RV systolic performance and its afterload, i.e. PA pressure [7]. Surrogate parameters to assess RV/PAc can easily be assessed in echocardiography, where RV/PAc is defined as the ratio of the tricuspid annular plane systolic excursion (TAPSE) and the systolic pulmonary artery pressure (sPAP). The latter is defined as the RV to right atrial pressure gradient (RV/RA gradient) plus central venous pressure (CVP), which can be estimated by echocardiographic assessment of the inferior vena cava (IVC) [8]. A low value of RV/PAc is considered pathological, i.e. RV dysfunction.

RV/PAc is increasingly investigated in a number of conditions and was shown to have prognostic relevance in patients with pulmonary hypertension [9], heart failure [10], and valvular heart disease including mitral [11] and tricuspid regurgitation [12, 13]. However, the use of RV/PAc for treatment decision in AS patients undergoing TAVI warrants further investigation [14, 15]. Moreover, data on RV/PAc in different flow types of AS, such as low-flow low-gradient (LFLG) AS, are lacking. This study analyses the prognostic value of RV/PAc and elucidates its role in different flow types of AS.

## Methods

### Study cohort, inclusion and exclusion criteria

All consecutive patients undergoing transfemoral TAVI for severe AS at our centre as part of the EVERY-Valve registry between 2018 and 2020 were assessed. Since RV to right-atrial pressure gradients are necessary for estimation of sPAP, only patients with tricuspid regurgitation greater than grade 0 could be included. Patients with insufficient echocardiography images were excluded from the analysis. Patients were split into two groups according to reduced and preserved RV/PAc values (Supplemental Figure S1).

Data were collected as part of routine documentation according to quality control requirements and the EVERY-Valve registry, which was approved by the ethics committee of Ludwig-Maximilians-University Munich (ethical code number 19–840). All data collection and analyses were performed according to the declaration of Helsinki. Follow-up data were collected by phone or outpatient-clinic visits at 30 days after TAVI and yearly thereafter as described before [3].

### Echocardiographic definition of aortic stenosis flow type

All patients have undergone a detailed transthoracic echocardiographic assessment prior to TAVI. Images were reassessed by independent echocardiographers according to current guidelines [16]. Aortic valve area was calculated using the continuity equation method. Continuous-wave and pulsed-wave Doppler echocardiograms were used to calculate stroke volumes and transvalvular gradients. Patients were split into groups according to AS flow types defined by mean transvalvular pressure gradient (dPmean), left-ventricular ejection fraction (LVEF), and SVi, as described before [3]: high gradient (HG, dPmean ≥ 40 mmHg), classical LFLG (dPmean < 40 mmHg and LVEF < 50%), paradoxical LFLG (dPmean < 40 mmHg, LVEF ≥ 50%, stroke volume index, SVi, ≤ 35 ml/m^2^), and normal-flow low-gradient (NFLG, dPmean < 40 mmHg, LVEF ≥ 50%, SVi > 35 ml/m^2^).

### Echocardiographic determination of RV/PAc

RV/PAc was defined as TAPSE divided by estimated sPAP. TAPSE was assessed either by M-mode with the cursor aligned along the tricuspid annulus systolic excursion or by a two-dimensional approach in a four-chamber or RV-focused view [17]. sPAP was obtained by adding the RV/RA pressure gradient to the estimated CVP. RV/RA gradients were obtained by integrating the tricuspid valve regurgitation peak velocity (Vmax) into the simplified Bernoulli equation ($RV/RA gradient=4\times {Vmax}^{2}$). CVP was estimated using IVC diameter (IVCd) and IVC respiratory variability (normal when > 50%) in accordance with guidelines and literature [8, 18]: IVCd < 21 mm and normal respiratory variability: CVP 3 mmHg, IVCd < 21 mm and unclear respiratory variability: CVP 5 mmHg, normal respiratory variability and unclear IVCd: CVP 5 mmHg, IVCd > 21 mm or reduced respiratory variability: CVP 8 mmHg, IVCd > 21 mm and reduced respiratory variability: CVP 15 mmHg.

### TAVI procedure and medication

Transfemoral access and local anaesthesia were used for all patients. Prosthesis choice and the performance of pre- and post-dilatation were left to the operator’s discretion. For peri-procedural anticoagulation, unfractionated heparin was used. Suture-mediated closure devices were used for access-site closure. In patients with an indication for an oral anticoagulation, this was continued after the procedure. All other patients were treated with 100 mg acetylsalicylic acid plus 75 mg clopidogrel for 3 months followed by lifelong 100 mg acetylsalicylic acid. If patients had an indication for anticoagulation and dual platelet inhibition, therapeutic regimens were conducted according to the guidelines.

### Endpoints

Primary endpoint of the analysis was all-cause mortality at 2 years. Further endpoints included cardiovascular mortality at 2 years and procedural and clinical outcome endpoints as defined by the Valve Academic Research Consortium (VARC) 3, including the two composite endpoints ‘technical failure’ and ‘device failure’ [19]. Subgroup analyses were performed for the four different AS flow types: HG, classical LFLG, paradoxical LFLG, and NFLG.

### Statistical analysis

Statistical analysis was performed with R, version 4.0.0 (RStudio Inc., Boston, MA, USA); graphs were designed with Prism for macOS version 9.5.1 (GraphPad Software, San Diego, CA, USA) and Adobe Illustrator version 26.5 (Adobe Inc., San Jose, CA, USA). Continuous variables are presented as median [interquartile range] or mean (± standard deviation). Shapiro–Wilk test was used for normality assessment. Categorial variables are presented as absolute numbers and percentages. Values were compared using Fisher’s exact, Chi-squared, Kruskal–Wallis, Wilcoxon, or Mann–Whitney tests as appropriate. For dichotomization of the cohort, a receiver operator curve (ROC) analysis for mortality employing Youden’s *J* statistic was used. A multivariate Cox proportional hazards model with backwards elimination was performed. Threshold for inclusion in the model was a *p*-value < 0.1 in univariate analyses. For comparison of statistical models, continuous net reclassification improvement (NRI) was used. Mortality analyses were performed by the Kaplan–Meier method and Log-rank test. A competing risk model was used to analyse cardiovascular death rates [20]. Generally, a *p*-value of < 0.05 was considered statistically significant.

## Results

A total of 1577 consecutive patients from 2018 to 2020 were screened (Supplemental Figure S1). Among 973 patients with tricuspid regurgitation grade 1 or above, 111 were excluded due to insufficient echocardiography images, mostly due to undeterminable RV/RA gradients. In total, 862 patients were included in the analysis. The ROC analysis resulted in a cut-off value of 0.512 mm/mmHg, which was close to the overall median RV/PA coupling value (0.511 [0.346–0.706] mm/mmHg). The study cohort was split into 433 patients with RV/PAc equal to or below the cut-off (reduced RV/PAc) and 429 patients above the cut-off (preserved RV/PAc).

### Baseline characteristics and procedural details

Clinical characteristics at baseline are presented in Table 1. Median RV/PAc was 0.345 [0.286–0.422] mm/mmHg in the reduced and 0.710 [0.594–0.857] mm/mmHg in the preserved group, respectively (*p* < 0.001). Patients in the reduced group were more often male, were numerically older, had significantly higher Society of Thoracic Surgeons (STS) scores, and had higher rates of chronic obstructive pulmonary disease, atrial fibrillation, coronary artery disease, and chronic kidney disease.

**Table 1:** Clinical characteristics at baseline

	Reduced (*N* = 433)	Preserved (*N* = 429)	*p* value
Male sex	234 (54.0%)	202 (47.1%)	**0.041**
Age (years)	82.8 [78.9–86.7]	81.8 [78.1–85.7]	0.067
Body mass index (kg/m^2^)	25.6 [23.0–28.1]	25.1 [23.1–27.9]	0.277
Body surface area (m^2^)	1.84 [1.69–1.98]	1.82 [1.67–1.96]	0.163
STS score	4.00 [2.51–6.00]	2.78 [2.00–4.00]	** < 0.001**
Diabetes mellitus type 2	130 (30.0%)	101 (23.6%)	**0.033**
Hypertension	399 (92.1%)	380 (88.6%)	0.076
Smoker (active or past)	98 (22.6%)	97 (22.6%)	0.994
Hypercholesterolaemia	194 (44.9%)	170 (39.9%)	0.138
Positive family history	52 (12.0%)	57 (13.3%)	0.573
Chronic kidney disease	232 (53.6%)	118 (27.5%)	** < 0.001**
COPD	60 (14.4%)	40 (9.7%)	**0.039**
Atrial fibrillation	181 (41.8%)	78 (18.2%)	** < 0.001**
Coronary artery disease	266 (61.4%)	229 (53.4%)	**0.017**
Prior MI	54 (12.5%)	41 (9.6%)	0.172
Prior PCI	131 (33.9%)	112 (28.9%)	0.141

Data are presented as number (percentage) or median [interquartile range]

*COPD*, chronic obstructive pulmonary disease; *MI*, myocardial infarction; *PCI*, percutaneous coronary intervention; *STS score*, Society of Thoracic Surgeons score

Groups differed in most echocardiographic characteristics (Table 2). Patients with reduced RV/PAc had lower LVEF and higher rates of relevant aortic, mitral, and tricuspid regurgitation.

**Table 2:** Echocardiographic characteristics at baseline

	Reduced (*N* = 433)	Preserved (*N* = 429)	*p* value
Aortic valve parameters			
AVA (cm^2^)	0.68 [0.55–0.85]	0.73 [0.61–0.90]	** < 0.001**
AVAi (cm^2^/m^2^)	0.37 [0.30–0.46]	0.41 [0.34–0.50]	** < 0.001**
dPmax (mmHg)	53.0 [40.0–68.5]	64.0 [49.0–74.0]	** < 0.001**
dPmean (mmHg)	33.0 [23.0–43.0]	40.0 [29.0–48.0]	** < 0.001**
SV (ml)	56.0 [46.8–68.0]	68.0 [57.0–82.0]	** < 0.001**
SVi (ml/m^2^)	31.0 [25.2–37.4]	37.9 [31.2–45.8]	** < 0.001**
Flow groups			** < 0.001**
High gradient	149 (37.8%)	221 (55.8%)	
Classical LFLG	116 (29.4%)	41 (10.4%)	
Paradoxical LFLG	88 (22.3%)	63 (15.9%)	
NFLG	41 (10.4%)	71 (17.9%)	
Valvular defects			
AI > 1	77 (18.0%)	48 (11.3%)	**0.005**
MI 3–4/4	35 (8.2%)	8 (1.9%)	** < 0.001**
TI > 1	200 (46.2%)	37 (8.6%)	** < 0.001**
Right ventricular parameters			
RV base diameter (cm)	4.80 [4.30–5.40]	4.50 [4.00–4.90]	** < 0.001**
RV mid-diameter (cm)	3.40 [3.00–4.00]	3.10 [2.80–3.60]	** < 0.001**
RV length (cm)	7.50 [7.00–8.10]	7.40 [6.80–8.10]	0.057
RV EDA (cm^2^)	24.3 [20.7–28.9]	21.8 [19.1–25.3]	** < 0.001**
RV ESA (cm^2^)	16.1 [12.9–19.8]	13.2 [11.0–15.9]	** < 0.001**
RV FAC (%)	34.0 [27.0–40.1]	38.7 [32.8–44.6]	** < 0.001**
TAPSE (mm)	17.0 [14.0–20.0]	23.0 [20.0–26.0]	** < 0.001**
RV/RA gradient (mmHg)	42.0 [35.0–51.0]	27.0 [23.0–32.0]	** < 0.001**
RV/PAc (mm/mmHg)	0.35 [0.29–0.42]	0.71 [0.59–0.86]	** < 0.001**
RA area (cm^2^)	34.5 [30.8–40.2]	24.0 [19.0–33.1]	** < 0.001**
RA volume (ml)	89.9 [63.6–121.6]	61.4 [48.5–87.2]	** < 0.001**
VCI diameter (cm)	1.99 [1.68–2.30]	1.65 [1.38–1.86]	** < 0.001**
Left-sided parameters			
Ejection fraction (%)	50.0 [40.0–55.0]	55.0 [53.0–58.0]	** < 0.001**
E (cm)	108.0 [87.9–132.0]	83.4 [65.6–106.0]	** < 0.001**
A (cm)	69.4 [47.2–102.0]	95.6 [72.8–115.0]	** < 0.001**
Medial E′ (cm)	5.44 [4.40–6.53]	5.00 [4.23–5.77]	**0.022**
Lateral E′ (cm)	7.90 [5.99–9.28]	6.50 [5.44–7.81]	** < 0.001**
LAVI (ml/m^2^)	54.0 [43.5–71.1]	41.7 [31.3–53.7]	** < 0.001**
LVIDd (cm)	4.90 [4.40–5.40]	4.50 [4.00–5.00]	** < 0.001**
IVSd (cm)	1.30 [1.10–1.40]	1.30 [1.10–1.50]	0.075
LVPWd (cm)	1.20 [1.00–1.40]	1.15 [1.00–1.30]	0.200

Data are presented as number (percentage) or median [interquartile range]

*AI*, aortic insufficiency; *AVA(i)*, aortic valve opening area (index); *dPmax*, maximum transvalvular gradient; *dPmean*, mean transvalvular gradient; *EDA*, end-diastolic area; *ESA*, end-systolic area; *FAC*, fractional area change; *IVSd*, interventricular septum thickness; *LAVI*, left atrial volume index; *LFLG*, low-flow low-gradient; *LVIDd*, left ventricular diastolic diameter; *LVPWd*, left ventricular posterior wall thickness; *MI*, mitral insufficiency; *NFLG*, normal-flow low-gradient; *RA*, right atrium; *RV*, right ventricle; *RV/PAc*, right ventricular to pulmonary artery coupling; *SV(i)*, stroke volume (index); *TAPSE*, tricuspid annular plane systolic excursion; *TI*, tricuspid insufficiency; *VCI*, inferior vena cava

Median valve prosthesis size used was slightly higher in the reduced than in the preserved group. All procedural details, including valve types, are shown in Supplemental Table S1.

### Long-term all-cause and cardiovascular mortality

**Fig. 1:**
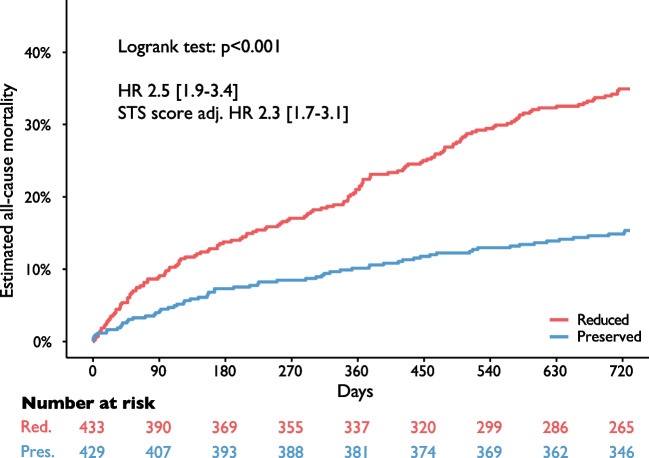
Kaplan–Meier estimates of 2-year all-cause mortality by RV/PAc groups. Patients with reduced right-ventricular to pulmonary artery coupling (RV/PAc) had significantly increased 2-year mortality (35.0% [95% confidence interval (95%CI), 30.3–39.3%] vs. 15.4% [95%CI, 11.9–18.7%], *p* < 0.001)

Follow-up rates were 99% and 94% at 1 and 2 years, respectively. Estimated 2-year mortality was significantly higher in the reduced group (35.0% [95% confidence interval (95%CI), 30.3–39.3%] vs. 15.4% [95%CI, 11.9–18.7%], hazard ratio (HR) 2.5 [1.9–3.4], *p* < 0.001, Fig. 1). Results were consistent when adjusting for STS score (adjusted HR, 2.3 [1.7–3.1]).

In a multivariable model, RV/PAc group prevailed as a predictor for 2-year mortality and was found to be stronger than its components, TAPSE and RV/RA gradient, or higher-grade tricuspid regurgitation (Supplemental Table S2).

Cardiovascular death accounted for a comparable fraction in both groups (67.4% vs. 65.2%, *p* = 0.715). The 2-year cardiovascular mortality was almost tripled in the reduced group (reduced 23.7% [95%CI, 19.4–27.8%], vs. preserved 8.5% [95%CI, 5.8–11.2%], HR 2.9 [2.0–4.2], *p* < 0.001, Supplemental Figure S2).

### Technical and clinical outcomes

No relevant differences in procedural outcomes were found according to the VARC-3 composite endpoint technical failure (3.7% for both, *p* = 0.979). Likewise, rates of the VARC-3 composite endpoint device failure at 30 days were comparable (reduced, 11.1% vs. preserved, 13.3%, *p* = 0.323), despite a relevant increase in 30-day mortality in the reduced group. Further differences were found for elevated dPmean at follow-up (more frequent in preserved group) and rate of stage 3 or 4 acute kidney injury (more frequent in reduced group). Detailed data are presented in Supplemental Table S3.

**Fig. 2:**
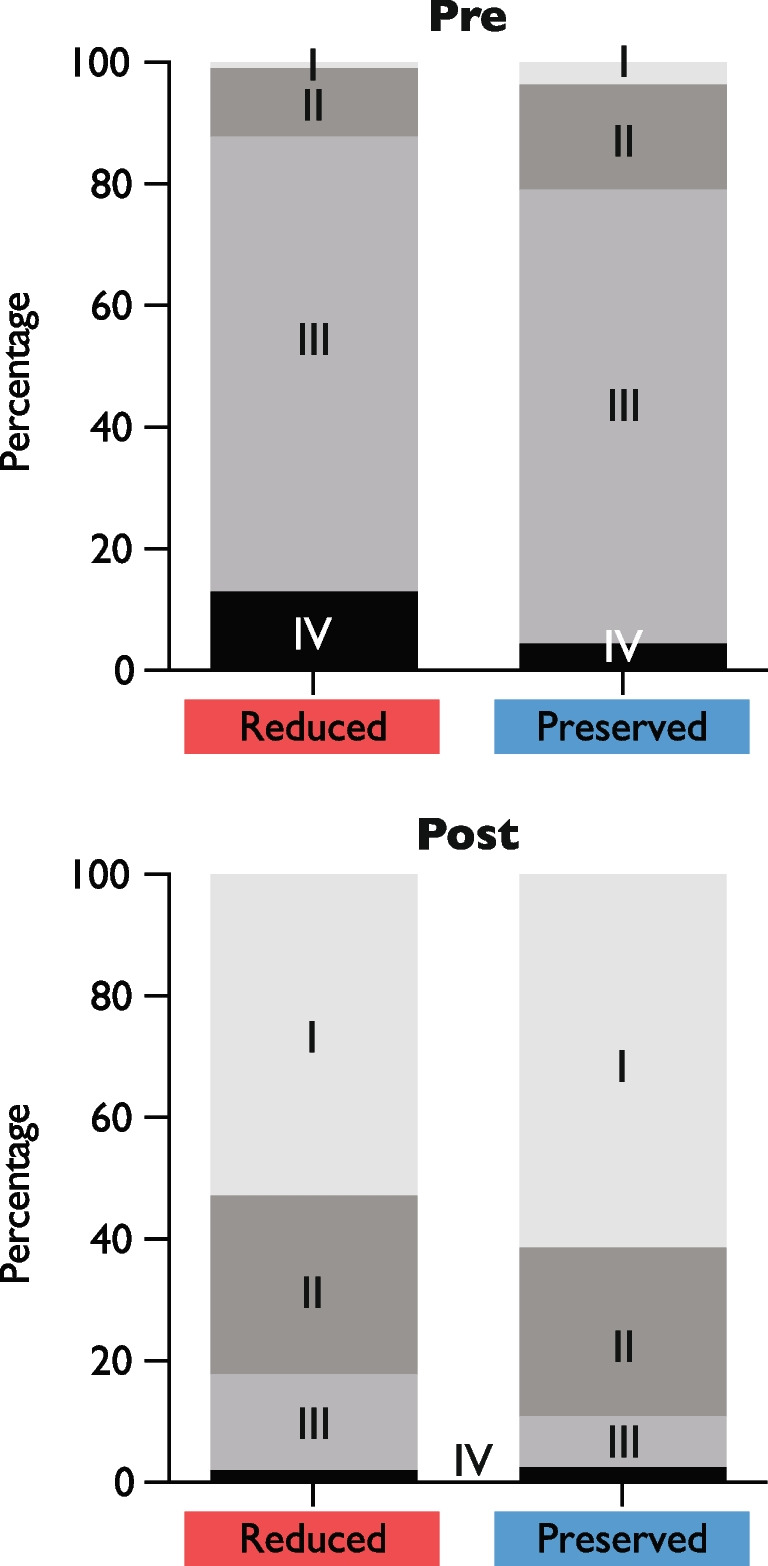
New York Heart Association (NYHA) functional class before and after TAVI. Bar chart showing distribution of NYHA functional classes pre and post TAVI by group. While NYHA functional classes differed before TAVI (*p* < 0.01), there was no significant difference after TAVI (*p* = 0.059)

Despite a trend to a more severe New York Heart Association (NYHA) functional class in patients in the reduced group at baseline, similar outcomes in both groups could be observed at latest available follow-up (Fig. 2). Moreover, similar fractions of patients in both groups improved by at least one class (reduced RV/PAc 76.1%, preserved RV/PAc 80.0%, *p* = 0.312).

### Subgroup analyses for different aortic stenosis flow types

**Fig. 3:**
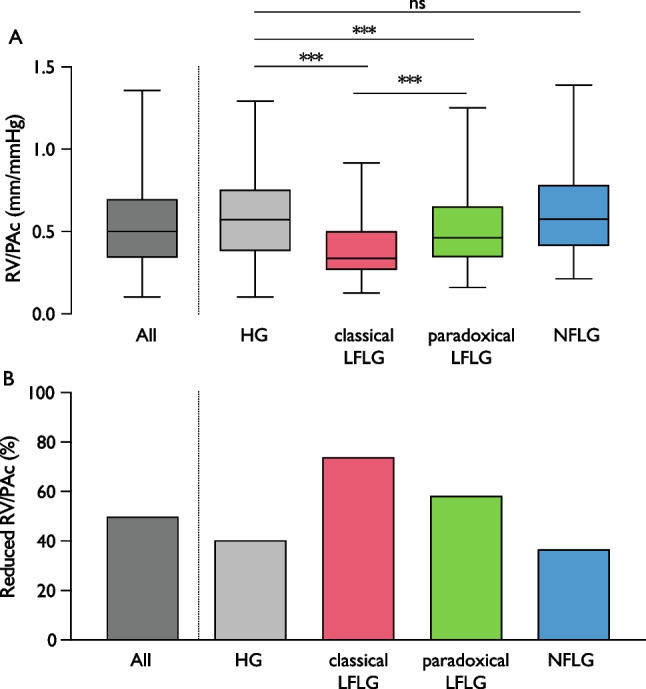
RV/PAc values by aortic stenosis flow type. **A** Values of right ventricular to pulmonary artery coupling (RV/PAc) differed between aortic stenosis flow types (*p* overall < 0.01): all patients, 0.511 [interquartile range (IQR), 0.346–0.706] mm/mmHg; high gradient (HG), 0.579 [IQR, 0.389–0.782] mm/mmHg; classical low-flow low-gradient (LFLG), 0.341 [IQR, 0.273–0.521] mm/mmHg; paradoxical LFLG, 0.462 [IQR, 0.346–0.642] mm/mmHg; normal-flow low-gradient (NFLG), 0.578 [IQR, 0.416–0.787] mm/mmHg. **B** Fraction of reduced RV/PAc was significantly different between groups (*p* overall < 0.01). Asterisks indicate level of significance (***p < 0.001; **p < 0.01; *p < 0.05; ns, not significant)

Data were stratified by AS flow types. Median RV/PAc values differed significantly between flow type groups: HG 0.58 mm/mmHg, classical LFLG 0.34 mm/mmHg, paradoxical LFLG 0.46 mm/mmHg, and NFLG 0.58 mm/mmHg, *p* < 0.01 (Fig. 3A). Accordingly, RV/PAc was classified as reduced in 40.3% (HG), 73.9% (classical LFLG), 58.9% (paradoxical LFLG), and 36.6% (NFLG), respectively (*p* < 0.01, Fig. 3B).

**Fig. 4:**
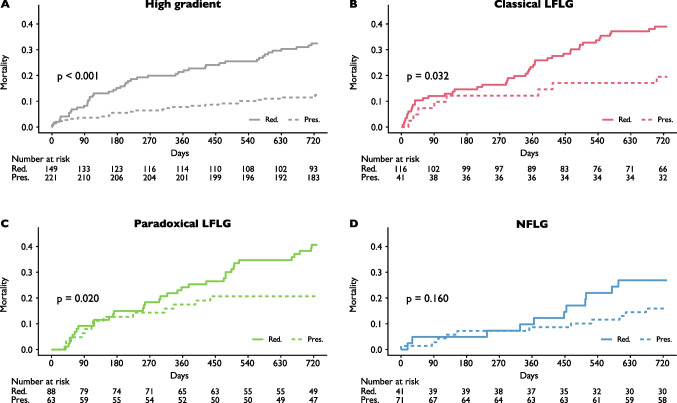
Mortality by RV/PAc according to aortic stenosis flow type. Kaplan–Meier curves estimating 2-year mortality according to aortic stenosis flow types. Dashed lines indicate preserved right ventricular to pulmonary artery coupling (RV/PAc). Mortality rates in patients with reduced RV/PAc were higher in patients with high gradient (**A**), classical low-flow low-gradient (LFLG) (**B**), and paradoxical LFLG (**C**) AS. In patients with normal-flow low-gradient (NFLG) (**D**), mortality rates did not differ statistically between reduced and preserved RV/PAc. Mortality rates were as follows: high gradient, estimated 2-year mortality rates, reduced RV/PAc, 32.5% [95% confidence interval (95%CI), 24.4–39.7%] vs. preserved RV/PAc 12.4% [95%CI, 7.9–16.7%]; classical low-flow low-gradient (LFLG), estimated 2-year mortality rates, reduced RV/PAc, 38.9% [95%CI, 29.4–47.2%] vs. preserved RV/PAc, 19.5% [95%CI, 6.4–30.8%]; paradoxical LFLG, estimated 2-year mortality rates, reduced RV/PAc, 40.7% [95%CI, 29.3–50.2%] vs. preserved RV/PAc, 20.6% [95%CI, 10.0–30.0%]; normal-flow low-gradient (NFLG), estimated 2-year mortality rates, reduced RV/PAc, 26.8% [95%CI, 11.9–39.2%] vs. preserved RV/PAc, 15.9% [95%CI, 6.8–24.1%]

In mortality sub-analyses, reduced RV/PAc was found to determine 2-year all-cause mortality among the subgroups of HG patients, paradoxical LFLG patients, and classical LFLG patients (Fig. 4A–C). Mortality rates in the small subgroup of patients with NFLG AS did not differ statistically despite a visual separation of the curves (Fig. 4D).

Mortality differences between AS flow types and RV/PAc groups were further analysed in a multivariate model incorporating the STS score to account for further differences in baseline characteristics. RV/PAc was found to be a stronger predictor for mortality than AS flow type (Table 3) and its addition to the model yielded incremental predictive value (continuous NRI 0.266 [95%CI, 0.172–0.343], *p* < 0.001).

**Table 3:** Multivariate model for prediction of 2-year mortality

	Odds ratio [95% confidence interval]	*p* value
Reduced RV/PAc	2.17 [1.58–3.00]	** < 0.001**
Classical LFLG vs. HG	1.19 [0.82–1.73]	0.372
Paradoxical LFLG vs. HG	1.42 [0.98–2.05]	0.062
NFLG vs. HG	0.92 [0.57–1.48]	0.729
STS score (per point increase)	1.07 [1.04–1.09]	** < 0.001**

Multivariate Cox model analysing the impact of right ventricular to pulmonary artery coupling (*RV/PAc*), Society of Thoracic Surgeons (*STS*) score, and aortic stenosis flow types on 2-year mortality. In this model, RV/PAc and the STS score prevail as predictors of 2-year mortality. Continuous net reclassification index (*NRI*) indicated an improvement of the predictive value of the model when RV/PAc was added to STS score and flow type (continuous NRI 0.266 [95%CI, 0.172–0.343], *p* < 0.001)

*HG*, high-gradient aortic stenosis; *LFLG*, low-flow low-gradient aortic stenosis; *NFLG*, normal-flow low-gradient aortic stenosis

### Mortality in patients without tricuspid regurgitation

**Fig. 5:**
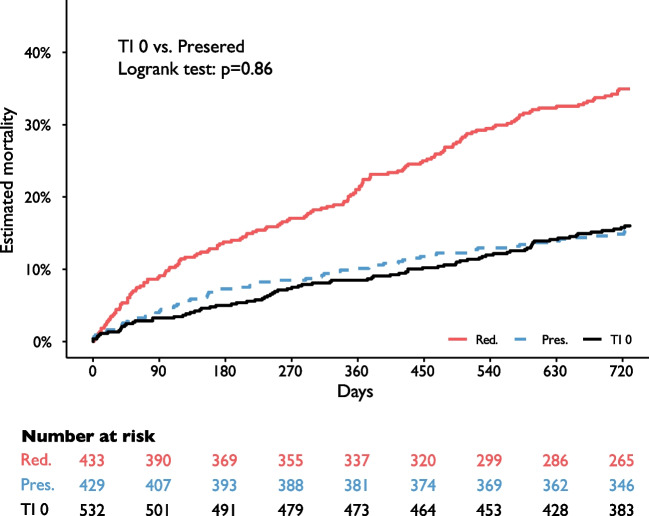
Mortality in RV/PAc groups and patients without tricuspid regurgitation. For assessment of right ventricular to pulmonary artery coupling (RV/PAc), estimation of systolic pulmonary artery pressure derived from right ventricular to right atrial gradients is necessary. Since this is not possible in echocardiography in patients without tricuspid regurgitation, these patients were not included in the analysis. As seen in these Kaplan–Meier curves, 2-year mortality in patients without tricuspid regurgitation (TI 0, black line 16.0% [95%CI, 12.8–19.1%]) is very similar to patients with preserved RV/PAc (blue dashed line, 15.4% [95%CI, 11.9–18.7%], *p* = 0.858)

Patients without tricuspid regurgitation in whom a RV/RA gradient cannot be obtained were not included in the primary analysis. In a separate mortality analysis, these patients (*n* = 532) were compared to the RV/PAc groups. Evidently, 2-year mortality in patients without tricuspid regurgitation (16.0% [95%CI, 12.8–19.1%]) was similar to patients with preserved RV/PAc (9% of which had tricuspid regurgitation grade 2 +) (*p* = 0.858, Fig. 5).

## Discussion

The present study is the largest and most concise evaluation of RV/PAc in TAVI patients [15, 18, 21–23]. Main findings include that (i) RV dysfunction, defined as reduced RV/PAc, is associated with a 2.5-fold increase in all-cause and a 2.9-fold increase in cardiovascular 2-year mortality; (ii) RV/PAc predicts mortality independent of AS flow type while (iii) RV/PAc values differ greatly between them; and (iv) RV/PAc is additive to established prognostic markers and (v) is stronger than its components.

### Relevance of RV dysfunction in aortic stenosis

RV dysfunction was shown to impact mortality in AS patients undergoing TAVI in a couple of studies [6, 24–26]. Numerous variables can be used for evaluation of right-sided heart disease. In several studies, RV dysfunction seemed to be of higher prognostic relevance than TR grade, which fits our results [21, 26, 27].

Right heart function is determined by dimensions of RV and RA, competency of the heart valves, and systolic and diastolic right ventricular function, and RV/PAc is a parameter that integrates several of these components. RV/PAc is a ratio and, as such, exceeded the prognostic impact of that of its components TAPSE and RV/RA gradient alone in our analysis, which is in accordance to the literature [21, 22]. An older very concise study assessed RV/PAc and could show that it remained as a significant mortality predictor in addition to age, atrial fibrillation, LVEF, and other common mortality predictors in a multivariate analysis, which is equivalent to our results [23]. In order to assess RV dysfunction, the utility of RV/PAc seems undeniable.

However, in a relevant number of patients, no RV/RA gradient can be obtained due to absence of tricuspid regurgitation, leading to high drop-out rates and unavailability of the parameter. Here, most of these patients (86%, data not shown) had normal TAPSE values. Mortality in this group was very similar to the preserved RV/PAc group (even though a relevant fraction of the latter had relevant tricuspid regurgitation). This implies that they can be assumed to have sufficient RV function and preserved RV/PAc, which strengthens the power of RV/PAc as a parameter.

### Pathophysiological mechanisms of RV dysfunction

RV dysfunction has been suggested as the ultimate result of a sequence of cardiac changes and adaptations induced by AS [4]. Yet, a relief of obstruction does not necessarily revoke right-sided damage. Often, an improvement in pulmonary pressure but not in RV function is witnessed, especially among patients with reduced RV/Pac [18, 21]. Assumably, RV damage may be a consequence of AS but can also be an independent entity, which has already been supposed by different authors [4, 21].

The relationship of LV and RV is rather complex. RV dilatation, tricuspid regurgitation, and, to some extent, RV dysfunction are certainly influenced by left-sided congestion. On the other hand, damage to oblique septal fibres shared by LV and RV as an early effect of AS on cardiac anatomy has been suggested as an explanatory mechanism for RV dysfunction [28]. Declining RV/PAc may be accompanying other changes of the cardiac structure as AS progresses, such as myocardial fibrosis, chamber dilatation, concomitant valve disease, or diastolic dysfunction.

### Implications of impaired RV/PAc

This raises the question whether patients with reduced RV/PAc would profit from earlier interventions, and ongoing studies on moderate AS may elucidate this. Surely, RV/PAc can help identify patients at risk for adverse events after TAVI who might profit from more frequent follow-up visits. Additionally, ways to positively influence RV dysfunction (pharmaceutical and structural) should be a focus of future investigations.

### Definition of RV/PAc cut-off value and association with comorbidities and AS flow type

In our study, we used a ROC analysis for mortality to define a cut-off value for grouping, which had been done by one other study so far (cut-off 0.36), while many other studies used the median [22, 23, 29]. In our study, median and ROC derived cut-off were almost identical. Older studies (with more high-risk patients) often have lower median RV/PAc values, e.g. 0.43 mm/mmHg in an analysis of 505 patients from 2011 to 2016 [23] or 0.39 mm/mmHg in selected AS patients with known RV dysfunction or pulmonary hypertension [29], illustrating that RV/PAc values are subject to the comorbidities of the patients. In a recent sub-analysis of the PARTNER 3 trial of low-risk patients (median 0.60 mm/mmHg) undergoing surgical aortic valve replacement (269 patients) or TAVI (301 patients), a cut-off value derived from Cox proportional hazards was used for the definition of reduced RV/PAc. They separated groups at 0.55 mm/mmHg, comparable to our results [21].

In our analysis, like in the literature, patients with reduced RV/PAc had more comorbidities (rates of atrial fibrillation and chronic kidney disease twice as high), higher STS scores, and worse LVEF. Accordingly, median RV/PAc values differed between AS flow types and were substantially lower in classical and paradoxical LFLG AS patients with more comorbidities, which resemble heart failure patients with reduced or preserved LVEF [3].

### RV dysfunction defined by RV/PAc determines prognosis

The main finding of our study, the extremely strong discriminatory value of RV/PAc for 2-year all-cause mortality, was comparable to the aforementioned studies and more recent publications [29, 30]. Notably, mortality differences between groups were not triggered by short-term procedural complications according to VARC-3, like in the PARTNER 3 sub-analysis. Nevertheless, in our data as in the PARTNER 3 sub-analysis, a symptomatic benefit could be derived irrespective of RV/Pac [21].

Mortality difference in patients with reduced RV/PAc prevailed in different AS flow types, underlining the relevance of the parameter. Similarly, an analysis of 65 LFLG patients published in 2016 was analysed for RV dysfunction and found it to be independently associated with mortality [31]. To our knowledge, no other study so far has analysed RV/PAc in context of AS flow types. Recently, the call for an analysis like this was raised by a group from France who evaluated RV/PAc in patients with preserved ejection fraction [32]. Increasing the knowledge on RV function in AS subtypes is of special importance since the pathophysiological understanding of NFLG and paradoxical LFLG AS in particular and the usefulness of TAVI in this context is still limited.

### Limitations

The present study has limitations inherent to retrospective registry analyses. Most importantly, the analysis was limited to the available echocardiography images, possibly entailing a selection bias and prohibiting evaluation of RV free wall strain or S′ instead of TAPSE, which could have yielded even more precise results [33]. In line with this, an analysis of follow-up echocardiography to assess changes in RV/PAc after TAVI was not deemed reasonable. Furthermore, the cut-off value for reduced RV/PAc was determined in a ROC analysis and may thus differ in other cohorts.

## Conclusions

In conclusion, the results from this large analysis identify RV/PAc as a strong mortality predictor in patients undergoing TAVI. RV/PAc is a stronger mortality predictor than its components, it improves prognostic accuracy when added to other risk factors in TAVI patients, and it retains its prognostic relevance in different AS flow types. Current guidelines disregard RV function. Future risk scores such as the STS score or the EuroSCORE should incorporate a more detailed assessment RV dysfunction.

## Supplementary Information

Below is the link to the electronic supplementary material.

Supplementary file1 (DOCX 183 KB)

## Data Availability

Data are available upon reasonable request to the corresponding author.

## Abbreviations

AS: Aortic stenosis
CVP: Central venous pressure
dPmean: Mean transvalvular pressure gradient
HG: High-gradient
IVC: Inferior vena cava
LFLG: Low-flow low-gradient
LVEF: Left ventricular ejection fraction
NFLG: Normal-flow low-gradient
RV: Right ventricular
RV/PAc: Right ventricular to pulmonary artery coupling
sPAP: Systolic pulmonary artery pressure
STS score: Society of Thoracic Surgeons score
SVi: Stroke volume index
TAPSE: Tricuspid annular plane systolic excursion
TAVI: Transcatheter aortic valve replacement
VARC-3: Valve Academic Research Consortium 3

## Declarations

The study has been conducted in accordance to the ethical standards laid out in the 1964 Declaration of Helsinki and its amendments. All patients gave their informed consent to the inclusion in the study.
