# Supplementary material for: Right ventricular to pulmonary artery coupling in patients with different types of aortic stenosis undergoing TAVI
Source: Clin Res Cardiol. 2024 May 15;114(2):227–38. doi: 10.1007/s00392-024-02457-8 (PMC11839695; doi:10.1007/s00392-024-02457-8)
Supplement: Supplementary file 1 — Supplementary file1 (DOCX 183 KB) [file 392_2024_2457_MOESM1_ESM.docx]

# Supplemental appendix

## Supplemental Table S1 Procedural details

|  | Reduced (N=433) | Preserved (N=429) | p value |
| --- | --- | --- | --- |
| Anulus area (cm^2^) | 4.49 [4.00-5.69] | 4.62 [3.84-5.37] | 0.865 |
| Anulus perimeter (mm) | 78.30 [74.08-89.38] | 79.10 [72.67-85.47] | 0.673 |
| Prosthesis type |  |  | 0.375 |
| Sapien | 299 (69.9%) | 281 (66.1%) |  |
| CoreValve | 57 (13.3%) | 57 (13.4%) |  |
| Other | 72 (16.8%) | 87 (20.5%) |  |
| Prosthesis sizes |  |  | 0.066 |
| <25 mm | 102 (23.8%) | 121 (28.5%) |  |
| 25-28 mm | 210 (49.0%) | 214 (50.5%) |  |
| >28 mm | 117 (27.3%) | 89 (21.0%) |  |
| Prosthesis size (mm) | 26.00 [25.00-29.00] | 26.00 [23.00-26.25] | **0.010** |
| Pre-dilatation (%) | 192 (44.3%) | 231 (53.8%) | **0.005** |
| Post-dilatation (%) | 28 (6.5%) | 29 (6.8%) | 0.862 |
| Concomitant percutaneous coronary intervention | 66 (15.2%) | 47 (11.0%) | 0.062 |

Data are presented as number (percentage) or median [interquartile range].

## Supplemental Table S2 Univariate and multivariate analysis with backwards elimination on 2-year mortality

|  | **Univariate analysis** | | **Multivariate analysis** | |
| --- | --- | --- | --- | --- |
| **Variable** | **Hazard ratio** | **p value** | **Hazard ratio** | **p value** |
| Reduced RV/PAc | 2.53 [1.89-3.39] | **<0.001** | 1.65 [1.15-2.36] | **0.006** |
| Male sex | 1.30 [0.99-1.70] | **0.056** | Eliminated in step 8 | |
| Age (years) | 1.04 [1.02-1.06] | **0.001** | 1.03 [1.01-1.06] | **0.006** |
| Body mass index (kg/m^2^) | 0.98 [0.95-1.01] | 0.199 |  |  |
| Body surface area (m^2^) | 0.89 [0.45-1.74] | 0.725 |  |  |
| Diabetes mellitus | 1.16 [0.86-1.55] | 0.332 |  |  |
| Hypertension | 0.89 [0.57-1.38] | 0.605 |  |  |
| Nicotine | 1.14 [0.83-1.55] | 0.421 |  |  |
| Hypercholestrolemia | 1.00 [0.76-1.31] | 0.995 |  |  |
| Positive family history | 0.85 [0.55-1.29] | 0.440 |  |  |
| COPD | 1.41 [0.97-2.05] | **0.068** | Eliminated in step 7 | |
| Chronic kidney disease | 2.42 [1.84-3.18] | **<0.001** | 1.76 [1.3-2.39] | **<0.001** |
| Atrial fibrillation | 1.71 [1.30-2.24] | **<0.001** | Eliminated in step 4 | |
| Coronary artery disease | 1.31 [0.99-1.73] | **0.057** | Eliminated in step 11 | |
| AVAi (cm^2^) | 0.41 [0.12-1.41] | 0.159 |  |  |
| dPmean (mmHg) | 0.98 [0.97-0.99] | **0.002** | Eliminated in step 6 | |
| SVi (ml/m^2^) | 0.96 [0.95-0.98] | **<0.001** | 0.98 [0.97-1] | **0.021** |
| EF (%) | 0.97 [0.96-0.98] | **<0.001** | Eliminated in step 10 | |
| AI2+ | 0.96 [0.65-1.42] | 0.843 |  |  |
| MI3+ | 1.47 [0.85-2.52] | 0.164 |  |  |
| TI2+ | 2.45 [1.87-3.21] | **<0.001** | 1.52 [1.1-2.09] | **0.010** |
| TAPSE (mm) | 0.93 [0.91-0.96] | **<0.001** | Eliminated in step 1 | |
| RV/RA gradient (mmHg) | 1.02 [1.01-1.03] | **<0.001** | Eliminated in step 9 | |
| RV base diameter (cm) | 1.49 [1.26-1.76] | **<0.001** | Eliminated in step 5 | |
| RV mid diameter (cm) | 1.63 [1.35-1.95] | **<0.001** | 1.28 [1.05-1.55] | **0.015** |
| RV length (cm) | 1.13 [0.96-1.32] | 0.131 |  |  |
| RA volume (ml) | 1.01 [1.00-1.01] | **<0.001** | Eliminated in step 3 | |
| VCI diameter (cm) | 2.02 [1.45-2.83] | **<0.001** | Eliminated in step 2 | |
| LAVI (ml/m^2^) | 1.01 [1.00-1.01] | 0.199 |  |  |
| LVIDd (cm) | 1.09 [0.92-1.30] | 0.322 |  |  |
| IVSd (cm) | 0.77 [0.46-1.30] | 0.333 |  |  |
| LVPWd (cm) | 1.06 [0.62-1.81] | 0.843 |  |  |

All parameters with p<0.1 in univariate analyses were included in the multivariate model. A backwards elimination was performed. Results after 10 steps are given in the table.

AI, aortic insufficiency; AVAi, aortic valve opening area (index); COPD, chronic obstructive pulmonary disease; dPmean, mean transvalvular gradient; IVSd, interventricular septum thickness; LAVI, left atrial volume index; LVIDd, left ventricular diastolic diameter; LVPWd, left ventricular posterior wall thickness; MI, mitral insufficiency; RA, right atrium; RV right ventricle; RV/PAc, right ventricular to pulmonary artery coupling; SVi, stroke volume (index); TAPSE, tricuspid annular plane systolic excursion; TI, tricuspid insufficiency; VCI, inferior vena cava.

## Supplemental Table S3 Procedural and clinical outcomes

|  | Reduced (N=433) | Preserved (N=429) | p value |
| --- | --- | --- | --- |
| **Technical failure composite endpoint** | 16 (3.7%) | 16 (3.7%) | 0.979 |
| Procedural death | 1 (0.2%) | 3 (0.7%) | 0.312 |
| Cardiac structural complication | 4 (0.9%) | 11 (2.6%) | 0.066 |
| Conversion to open surgery | 1 (0.2%) | 3 (0.7%) | 0.312 |
| Prosthesis dislocation | 2 (0.5%) | 1 (0.2%) | 0.568 |
| Second valve prosthesis required | 2 (0.5%) | 3 (0.7%) | 0.646 |
| Immediate vascular surgery/intervention | 8 (1.8%) | 5 (1.2%) | 0.411 |
| **Device failure (30d) composite endpoint** | 48 (11.1%) | 57 (13.3%) | 0.323 |
| All-cause mortality (30d) | 17 (3.9%) | 7 (1.6%) | **0.041** |
| Elevated dPmean (30d) | 4 (0.9%) | 15 (3.5%) | **0.010** |
| Relevant PVR (30d) | 14 (3.2%) | 24 (5.6%) | 0.091 |
| Vascular surgery/intervention (30d) | 8 (1.8%) | 6 (1.4%) | 0.602 |
| Stroke rate (30d) | 5 (1.2%) | 9 (2.1%) | 0.273 |
| BARC Type 3 bleeding (30d) | 62 (14.3%) | 81 (18.9%) | 0.072 |
| AKI stage 3 or 4 (30d) | 20 (4.6%) | 4 (0.9%) | **0.001** |
| Permanent pacemaker implantation (30d) | 59 (13.6%) | 62 (14.5%) | 0.727 |
| Early MI at 48 h | 2 (0.5%) | 5 (1.2%) | 0.250 |

Data are presented as number (percentage).

AKI, acute kidney injury; BARC, Bleeding Academic Research Consortium; MI, myocardial infarction; PVR, paravalvular regurgitation

## Supplemental Figure S1 Study flow chart


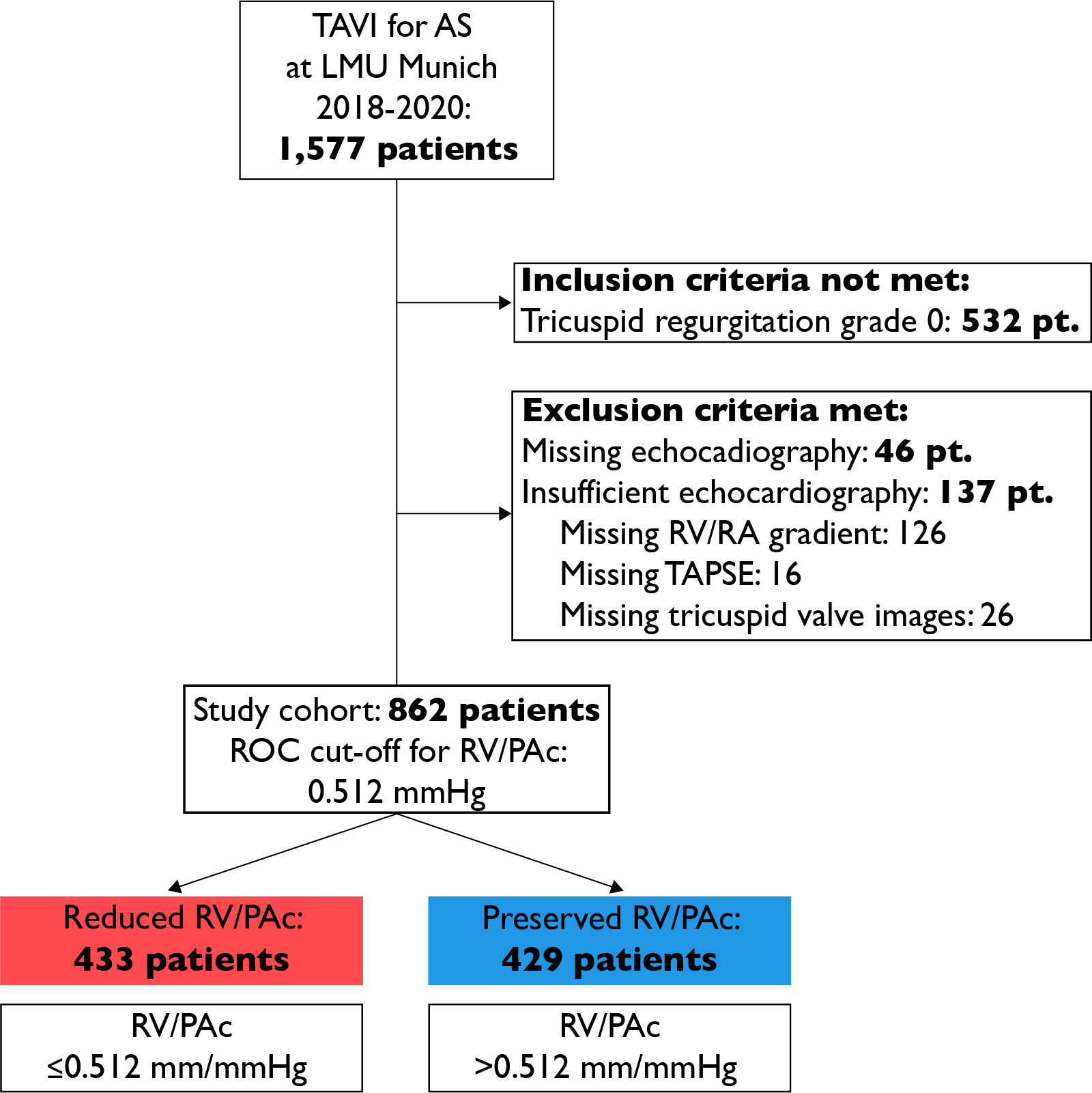


All patients undergoing transcatheter aortic valve implantation (TAVI) at LMU Munich between 2018 and 2020 were assessed. Right ventricular to pulmonary artery coupling (RV/PAc) was defined as the ratio of tricuspid annular plane systolic excursion (TAPSE) to estimated systolic pulmonary artery pressure (sPAP) obtained in echocardiography. Patients without tricuspid regurgitation were not included due to impossibility to acquire right ventricular/right atrial pressure gradients necessary to calculate RV/PAc. The study cohort was split according to a cut-off value for RV/PAc (0.5119 mm/mmHg) obtained from a receiver operator curve analysis.

## Supplemental Figure S2 Kaplan-Meier curve estimating 2-year cardiovascular mortality


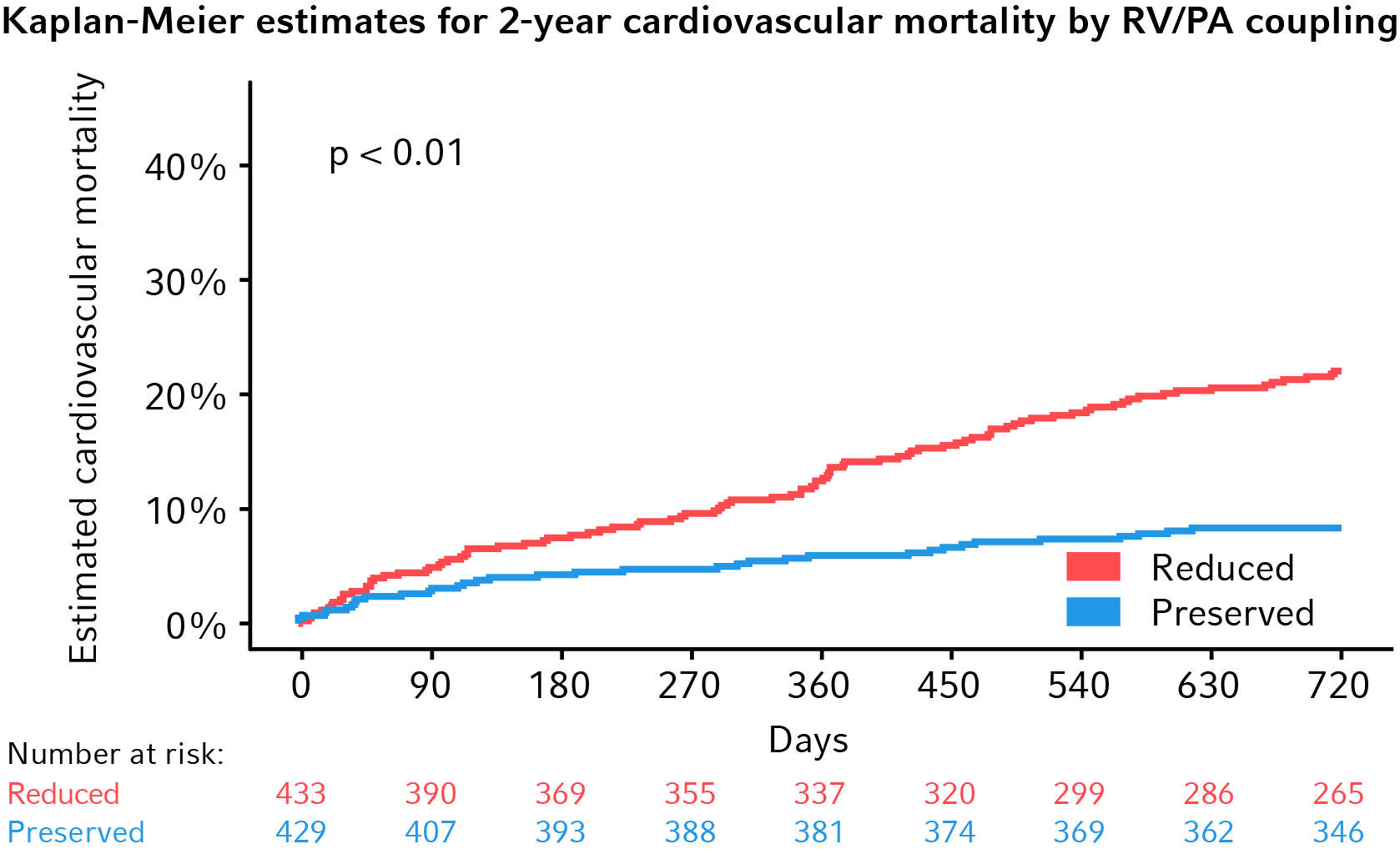


Kaplan-Meier curves estimating 2-year cardiovascular mortality show a significant difference between patients with preserved and reduced right ventricular to pulmonary artery coupling (RV/PAc). Cardiovascular mortality rates were: reduced, 23.7% [95% confidence interval, 95%CI, 19.4-27.8%], vs. preserved, 8.5% [95%CI, 5.8-11.2%]. Cardiovascular death was found in 67.4% and 65.2% of all deaths in reduced and preserved RV/PAc respectively.
